# Gene conversion in the rice genome

**DOI:** 10.1186/1471-2164-9-93

**Published:** 2008-02-25

**Authors:** Shuqing Xu, Terry Clark, Hongkun Zheng, Søren Vang, Ruiqiang Li, Gane Ka-Shu Wong, Jun Wang, Xiaoguang Zheng

**Affiliations:** 1 Beijing Institute of Genomics of Chinese Academy of Sciences, Beijing Genomics Institute, Beijing Proteomics Institute, Beijing 101300, China; 2 Department of Electrial Engineering and Computer Science, University of Kansas, Lawrence, KS 66046, USA; 3 Department of Biochemistry and Molecular Biology, University of Southern Denmark, DK-5230, Odense M, Denmark; 4 Research Unit for Molecular Medicine, Aarhus University Hospital and Faculty of Health Sciences, DK-8200 Aarhus N, Denmark; 5 Department of Biological Sciences and Department of Medicine, University of Alberta, Edmonton, Alberta, T6G 2E9, Canada; 6 The Institute of Human Genetics, University of Aarhus, DK-8000 Aarhus C, Denmark; 7 Kunming Institute of Zoology, Chinese Academy of Sciences, Kunming, 650223, China; 8 Graduate University of Chinese Academy of Sciences, Beijing, 100049, China; 9 Current address: Institute of Integrative Biology, ETH Zurich, 8092, Switzerland

## Abstract

**Background:**

Gene conversion causes a non-reciprocal transfer of genetic information between similar sequences. Gene conversion can both homogenize genes and recruit point mutations thereby shaping the evolution of multigene families. In the rice genome, the large number of duplicated genes increases opportunities for gene conversion.

**Results:**

To characterize gene conversion in rice, we have defined 626 multigene families in which 377 gene conversions were detected using the GENECONV program. Over 60% of the conversions we detected were between chromosomes. We found that the inter-chromosomal conversions distributed between chromosome 1 and 5, 2 and 6, and 3 and 5 are more frequent than genome average (Z-test, P < 0.05). The frequencies of gene conversion on the same chromosome decreased with the physical distance between gene conversion partners. Ka/Ks analysis indicates that gene conversion is not tightly linked to natural selection in the rice genome. To assess the contribution of segmental duplication on gene conversion statistics, we determined locations of conversion partners with respect to inter-chromosomal segment duplication. The number of conversions associated with segmentation is less than ten percent. Pseudogenes in the rice genome with low similarity to *Arabidopsis *genes showed greater likelihood for gene conversion than those with high similarity to *Arabidopsis *genes. Functional annotations suggest that at least 14 multigene families related to disease or bacteria resistance were involved in conversion events.

**Conclusion:**

The evolution of gene families in the rice genome may have been accelerated by conversion with pseudogenes. Our analysis suggests a possible role for gene conversion in the evolution of pathogen-response genes.

## Background

Gene conversion involves a non-reciprocal transfer of information between two homologous genes where one segment replaces nucleotides in its corresponding homolog. Gene conversion is generally considered a homogenization force on the genome, although it has two distinct consequences. In homogenization, gene conversion causes concerted evolution in gene families through reciprocal exchange of sequence between paralogs [1]. However, diversification can occur, for example, when a pseudogene or otherwise unexpressed gene segment is transferred into another, functioning gene. Alteration of gene function through diversification can have advantageous consequences, such as in immune system diversification involving the major histocompatibility complex genes [2-4].

In eukaryotes, gene conversion has been classified into two types based on the conversion targets; one involves allele conversion and the other involves repeated genes. Conversion between alleles occurs at the same loci on sister-chromatids or between homologous chromosomes. Conversion events between repeated genes can occur at different loci on the same chromatid, sister-chromatids, homologous chromosomes, or non-homologous chromosomes [5]. These events leave signatures in genome sequences that are detectable through specialized statistical analysis. In this study, we use such statistical methods with genome sequence data and annotations to study the gene conversion history of multigene families in the rice genome.

Genome wide searches for gene conversion events between paralogs have been performed in yeast [6], *Caenorhabditis elegans *[7], and mouse and rat [8]. Significant evidence for gene conversion events has been detected on human chromosomes 21 and Y [9-12]. Analyses of selected regions of the *Arabidopsis thaliana *genome suggest that the divergence during the process of gene evolution is affected by gene conversion [13,14]. However, prior to our work, no genome-wide conversion analysis has been reported for plants. As a result, little is known about how gene conversion influences the evolution of multigene families in plant genomes.

The rice genome has evidence of ancient whole genome duplication, as well as recent chromosomal and segmental duplication [15,16]. Because of the increase in paralogs through duplication, the rice genome may have undergone potentially many gene-conversion and unequal-crossover events in its evolution. Studies of these events can enhance our understanding of evolutionary processes behind multigene families in the rice genome. Toward this end, we have mined the rice genome database [16] for gene conversion traces.

## Results

### Number and length of gene conversions detected in the rice genome

**Figure 1:**
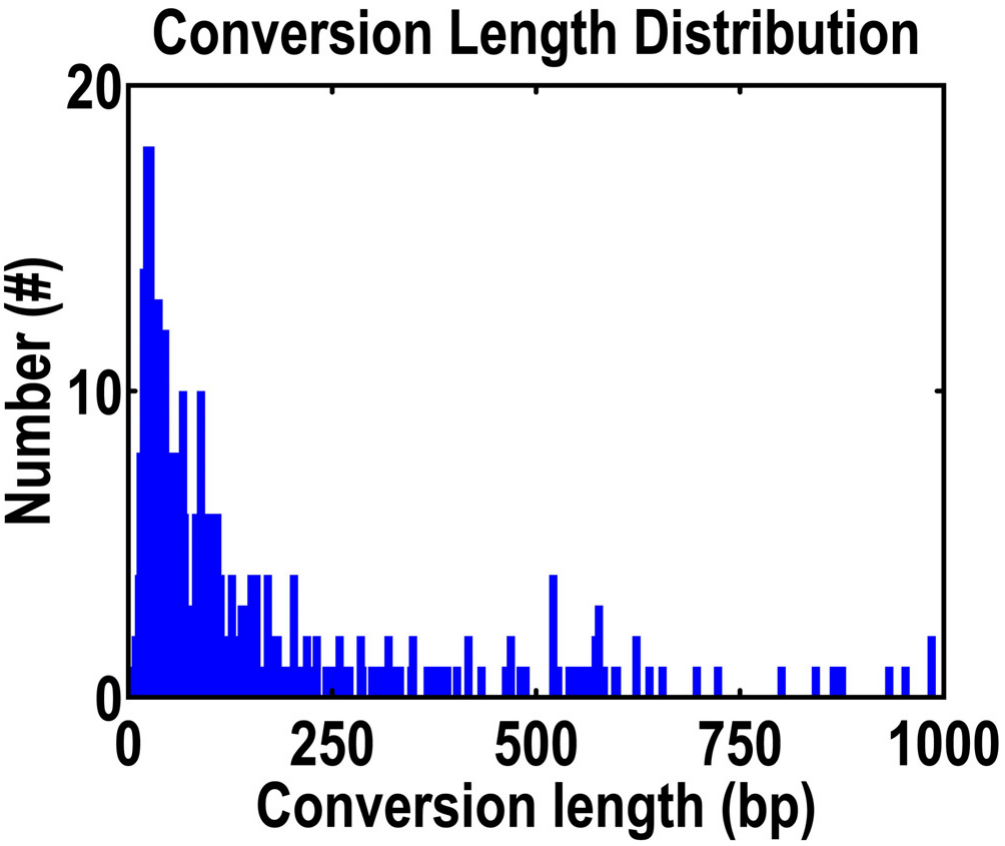
**Distribution of gene conversion regions in the rice genome**. Sixty-nine percent of all regions are shorter than 100 nucleotides. The number of conversions decreases as conversion length decreases.

We analyzed a dataset of 626 multigene families, each family with at least three paralogs. In a total of 5274 genes, we detected 377 gene conversion events involving 513 genes in 189 families. Approximately 66% of these conversions involved sequences shorter than 100 nucleotides (Figure 1). The number of conversions identified by the detection algorithm declines rapidly as sequence length approaches zero. (See Methods for a description of the detection algorithm.) The average length of all conversions is 130 nucleotides and ranges from 4 to 1237 nucleotides. In general, the length distribution of converted regions in the rice genome is similar to those found in other species [6,7]. Short conversions with fewer than about 10 nucleotides are usually considered to be artifacts (Stanley Sawyer, personal communication). Our analysis included six conversion events involving match lengths with less than 10 nucleotides; although these six events have low reliability, their presence does not influence the interpretation of our results.

Gene conversion with pseudogenes may accelerate gene family evolution; in this model, pseudogenes are postulated as a source of genetic information. The introduction of genetic material from pseudogenes may lead to higher divergence in orthologs between rice and related species. To test this hypothesis, we established rice gene families as having either low similarity (LS) or high similarity (HS) to *Arabidopsis thaliana*. HS gene families are defined as having statistically significant sequence similarity to *Arabidopsis *genes. Conversely, LS gene families have low similarity to *Arabidopsis*. It follows that LS gene families are more likely to be rice specific.

We categorized the 377 conversions detected in 50 LS and 327 HS gene families as described in Methods. Among the 377 conversion events, we identified those conversions involving pseudogenes in LS and HS families. The fraction of pseudogenes was approximately 56% in the LS families and 21% in the HS families (Table 1).

**Table 1:** Genes with low similarity to *Arabidopsis *were found to have more gene-conversion events than genes with high similarity. * Fisher's Exact Test P value < 0.01.

	**Total**	**with pseudogenes**	**Percent**
**LS**	50	28*	56.0%
**HS**	327	70	21.4%

To rule out effects from assembly artifacts on our study, we performed a similar analysis on the *japonica *rice genome published by The International Rice Genome Sequencing Project (IRGSP) [17]. The gene conversion characteristics were indistinguishable between the two assemblies (data not shown).

### Distribution of conversion events on the chromosomes

Our analysis of the rice genome detected 513 genes likely to be involved in 377 conversion events. To determine the distribution of gene conversion across the genome, we mapped the converted genes to chromosomes (Table 2). We were also interested in chromosomes with more conversions than the average number of conversions per unit length. To assess this, we estimated that the average number of conversions per million nucleotides for the entire genome was ~1.361. Based on the average frequency of 1.361 conversions per megabase, the number of conversions was found to be relatively uniform as a function of length for each chromosome. In the 377 events, 148 conversion pairings occurred within the same chromosome (~39%) as intra-chromosomal conversions; 229 conversion events occurred between chromosomes (~61%) as inter-chromosomal conversions.

**Table 2:** The number of genes involved in conversions on each chromosome compared to the estimated number based on chromosome length. The estimated number is the total number of conversions in the genome divided by the genome sequence length.

**Chromosome**	**Genes with conversions**	**Estimated genes with conversions**
Chr01	70	64.3
Chr02	45	51.9
Chr03	66	57.0
Chr04	37	47.5
Chr05	47	42.5
Chr06	49	44.8
Chr07	41	39.1
Chr08	65	41.4
Chr09	27	29.8
Chr10	28	30.9
Chr11	20	31.5
Chr12	18	32.4

Total	513	513

From a total of 3844 gene pairs in the 626 multigene families, 2903 pairs were located on different chromosomes, with 941 pairs co-located on the same chromosome. It follows that the proportion of inter-chromosomal pairs involved in conversions was ~8% (229/2903), and intra-chromosomal pairs involved in conversions was ~16% (148/941). Although the number of inter-chromosomal conversion events was higher than intra-chromosomal conversion, the inter-chromosomal fraction was lower with respect to the potential for conversion based on total gene pairs. Thus, candidate pairs on the same chromosome apparently result in a higher likelihood for gene conversion. This does not necessarily represent an intrinsic bias for intra-chromosomal gene conversion; alternatively, it may represent an opportunistic positioning for gene conversion to occur.

**Figure 2:**
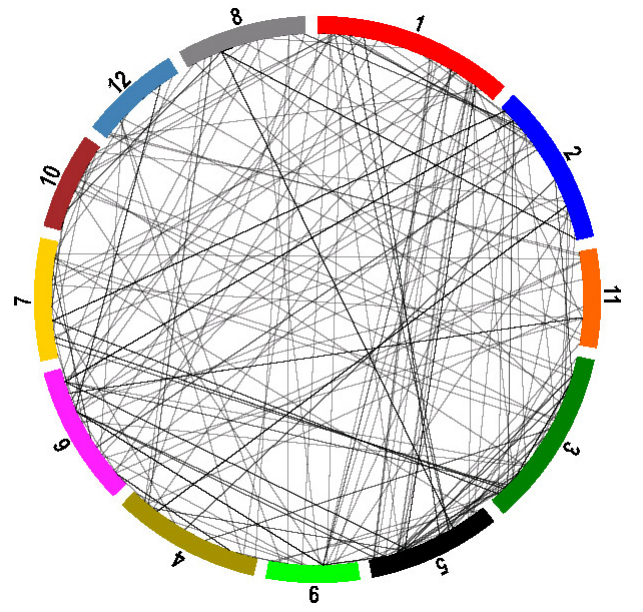
**Gene conversion distribution on the twelve rice chromosomes**. The two genes in each gene pair identified in a conversion are connected with a line. The line thickness indicates the frequency of the gene conversion between the corresponding chromosomes. The highest inter-chromosomal conversion frequencies were detected between chromosomes 1 and 5, 2 and 6, and 3 and 5.

The 229 inter-chromosomal conversion events are distributed non-uniformly among the twelve chromosomes of the rice genome. The conversion events are significantly more frequent between chromosomes 1 and 5, 2 and 6, and 3 and 5 than the average (Z-test, P < 0.05). The conversion distributions are shown in Figure 2 for each chromosome.

To determine the extent of segmental duplication on the observed conversion events, we compared conversion data with duplicated segments in the Beijing *indica *assembly [16]. For all 229 inter-chromosome conversion events, only 21 out of 229 (~9%) inter-chromosome conversion events were involved in the segmental duplications.

### Conversion bias based on sequence similarity and orientation

**Figure 3:**
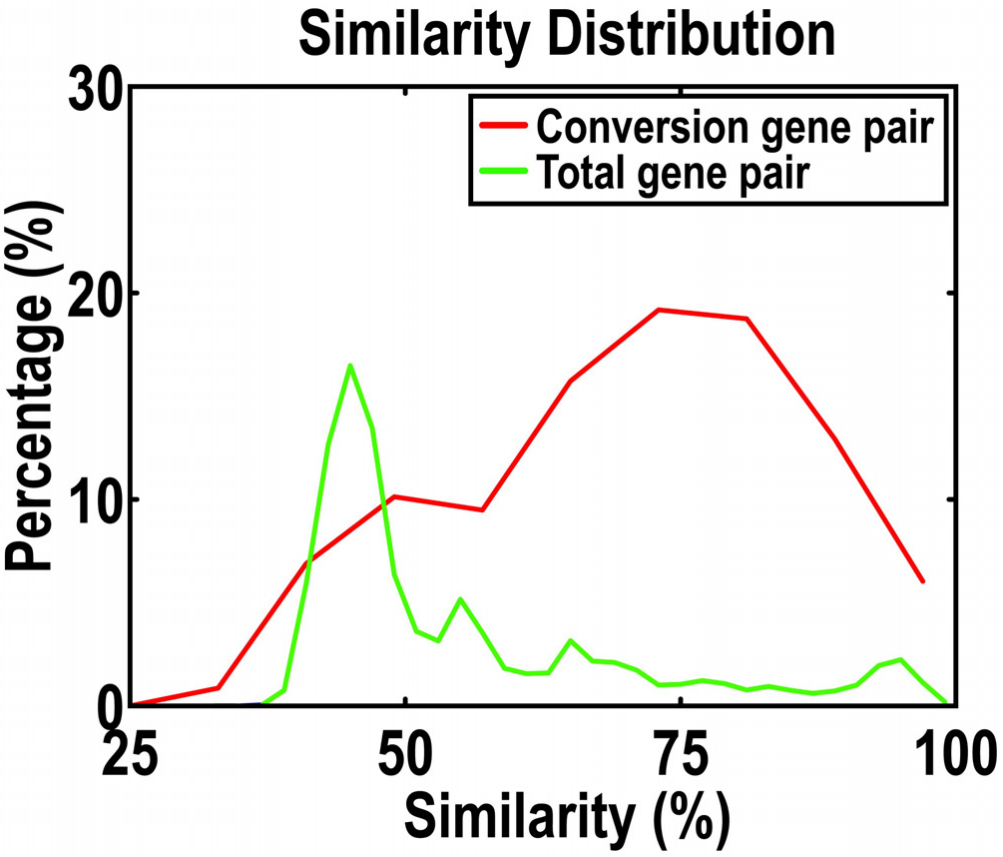
**Gene conversions are favored between similar genes**. Sequence similarities were calculated for homologs involved in gene conversions and for all homologs.

To compare the sequence similarity of gene pairs involved in conversions to gene pairs not involved in conversions, we aligned entire gene sequences as described in Methods. The fraction of gene pairs involved in conversion events had significantly higher sequence similarity compared to all gene pairs (Figure 3). To test if the converted regions themselves influenced the higher similarity in the conversion group, we also calculated similarities of genes in conversion pairs, omitting the converted regions from the analysis. No significant difference was found between before and after omitting the converted regions (data not shown); the higher sequence similarity of gene pairs involved in conversion was not only a feature of the converted regions. In fact, most gene pairs not involved in conversions share statistically significant similarities in about 30–60% of their sequences with an average sequence similarity of 45%. In contrast, gene pairs involved in conversions exhibit a greater sequence similarity in the range of 60–80%. These results are consistent with findings that gene conversion is favored between similar genes [18].

**Figure 4:**
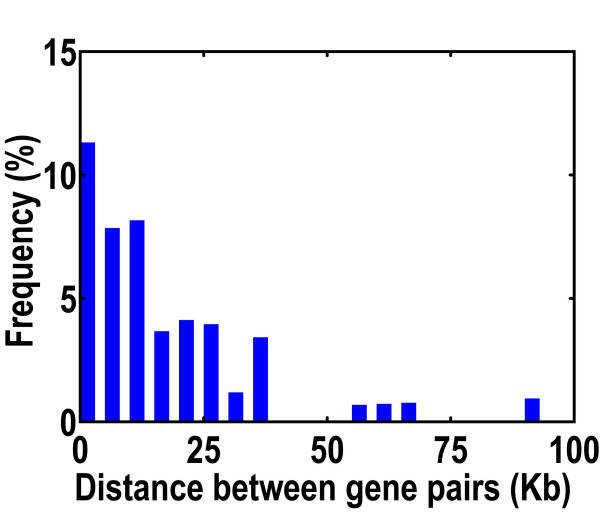
**Intra-chromosomal gene conversions frequencies**. The frequency of an intra-chromosomal conversion decreases as the distance between the gene pair increases.

For the 148 intra-chromosomal conversions, the conversion frequency decreased with the physical distance between gene pairs along the chromosome (Figure 4). The gene pairs separated by less than 5 kb demonstrated the highest conversion frequency; these could be considered closely linked genes. For gene pairs separated by more than ~35 kb, conversion was infrequent.

The directions between gene pairs were also examined. Among the 3844 gene pairs, 2172 had the same direction and 1672 had reverse directions. In the 377 conversion events, 277 (60%) occurred between gene pairs with the same direction (See details in Additional File 1). The proportions of conversions with same-direction gene pairs (227/2172 = ~10%) and reverse-direction gene pairs (150/1672 = ~9%) were similar. The larger number of gene conversion events in the same direction coincides with the larger number of gene pairs in the same direction; however, from our data we cannot determine whether the conversion bias is an intrinsic preference.

### Evolutionary selection correlated to gene function

To determine if gene pairs involved in conversions are subject to evolutionary selection pressure, synonymous substitution rates (Ks) and non-synonymous substitution rates (Ka) were used. The Ka/Ks ratio can reflect the selection pressure between gene pairs caused by evolutionary processes. We calculated and compared the Ka/Ks ratios for two groups: (1) the closest homologs in each multigene family where at least one homolog was involved in gene conversion and (2) all close homologs in each multigene family (see Methods). The Ka/Ks profiles for the two groups were indistinguishable (data not shown).

We assessed the function of genes involved in conversions using the protein *nr *database at NCBI [19]. Although the function of many genes is presently unknown, we identified approximately14 gene families involved in conversions to be related to disease or bacteria resistance. These include genes coding for phospholipase D, cytochrome P450, receptor-like kinase and receptor kinase-like proteins. Some conversions were also found in related *Arabidopsis *gene families [13,20]. (See details in Additional File 1). The highest conversion frequency was found in the phospholipase D (AK070203) gene family. Phospholipase D has been identified as an enzyme generating secondary messengers in plants, triggering defense against bacterial attacks [21].

## Discussion

The proliferation of duplications during the evolution of the rice genome may have increased the potential for gene conversion and crossover events within multigene families through an increase in donor sequences. In our analysis of rice, the likelihood for gene conversion was found to be greater between pairs on the same chromosome than pairs on different chromosomes, even though more pairs were found for the latter case. The large number of duplicated repeats between chromosomes provides numerous opportunities for inter-chromosomal gene conversion. That only ~9% of gene conversion occurred between pairs involved in inter-chromosome segmental duplications indicates that the observed conversions were primarily from other sources.

Our analysis considers fragments with uncharacteristically high similarity as candidates for gene conversion. High-similarity between fragments may also be caused by strong stabilizing selection. However, the fragments identified in gene conversion events are situated in spans of sequence flanked by sequence with low similarity. The low-similarity context suggests gene conversion and unequal crossing over as possible explanations for the high-similarity inner fragments.

We mapped all identified conversions onto the rice genome sequence (Figure 2). The most frequent conversions were between chromosomes 1 and 5, 2 and 6, and 3 and 5. Our data show a decrease in intra-chromosomal gene conversion frequency as the distance between genes increase. This distance dependence corresponds to previous genome-wide studies of yeast [6,7]. In *C. elegans*, a high proportion of conversion was observed between tandemly duplicated members of gene families [7]. The higher conversion frequency between genes with short separation on the same chromosome may be a consequence of a relationship between conversion and recombination. In *Arabidopsis*, it was found that the upper limit of pairwise distance between genes involved in conversion is 40 kb [13]. This is similar to the value we found in the rice genome (Figure 4).

Conversions involving pseudogenes could accelerate gene family evolution, and may accelerate divergence of some gene families relative to their orthologs. In this study, we found that pseudogenes are more prone to participate in gene conversion in the LS gene families than in HS gene families in the rice genome. Thus, conversion with pseudogenes in LS gene families may contribute to the acceleration of LS gene evolution. The rationale behind this is linked to the susceptibility of pseudogenes to accumulate mutations more rapidly than expressed genes, which may then be transferred to conversion partners. This may occur where pseudogene fragments are recruited into functional genes. By definition, LS genes have low similarity with *Arabidopsis*, which means LS gene families are potentially rice-specific. Based on this, we can postulate that inter-conversion with pseudogenes may be a source of rice-specific genes. A similar mechanism is suspected for some human speciation events [22]. Moreover, our findings support the view that pseudogenes contain potential material for new genes [23].

The question remains whether genes involved in conversions are subject to selective pressure that differs from non-converted genes. A mechanism has been suggested that favors selection of some gene clusters in the tomato plant exhibiting traces of gene conversion and conferring disease resistance [24]. But does this occur in rice and is it genome wide? We calculated Ka and Ks values and their ratios for groups of gene with and without conversions – no differences were observed between the two groups. These similar Ka/Ks ratios may indicate that the genes involved in conversions were not subject to significant selective pressure. The indistinguishable Ka/Ks ratios of gene pairs involved in conversion imply that the genome-wide gene conversions were not tightly linked to selection pressure in the rice genome. As suggested by Mondragon-Palomino and Gaut, these indistinguishable Ka/Ks ratios may be a result of both methodological and biological influences [13]. Inter-conversions through recombination and gene conversion may also influence the accuracy of Ka/Ks analysis [25]. Although the co-occurrence of gene conversion and positive selection has been found in some studies, there is evidence that gene conversion is independent of positive selection [13,26].

The diversification within gene families could be caused by conversion with variant paralogs [27]. This mechanism has been widely observed in mammalian immune systems [2,28,29]. In tomato and *Arabidopsis*, gene conversion has been detected in genes related to disease and bacteria resistance [13,24]. Our genome-wide analysis of rice identified at least fourteen such genes potentially influenced by gene conversion events. Some of these genes have counterparts in *Arabidopsis *[13], while others were specific to rice. Our results contribute to the view that intergenic gene conversion can create variety within a gene family, providing a mechanism for the adaptive evolution, such as disease resistance [3]. The diversified paralogs conferring disease resistance would be advantageous for adaptive reorganization and response to various diseases or bacteria.

## Conclusion

We have detected 377 gene conversion events in the rice genome. The overall characteristics of gene conversion in the rice genome suggest influences by extensive duplication events throughout the evolution of rice. Our data further suggest that conversion with pseudogenes may have accelerated the evolution of multigene families. In particular, the adaptive evolution of disease resistance in rice may have been significantly influenced by gene conversion.

## Methods

### Gene families

The initial 28,469 full-length rice cDNA sequences were downloaded from RIKEN and FAIS centers [30]. We aligned these cDNAs to the *Oryza sativa indica *genome sequence using BLAT [31]. We removed the following sequences: redundant genes, namely those that are the smaller cDNA of two alignments with overlap of at least 100 bp; unlikely protein coding genes with open reading frames less than 100 amino acids; and those sequences with more than 10% transposon-like content identified by using RepeatMasker with RepBase [32]. Eventually, 13,089 reliable protein-coding genes were obtained [33] and used as consensus sequences.

Paralogs in the rice genome (Indica 9311) were identified based on similarity with the 13,089 reference protein sequences using BLAST [34]. The pipeline used to identify paralogs is similar to the FGF approach [35]. The reference protein sequence was defined as the consensus sequence for each family. The members of gene families were defined by alignments with at least 80% similarity over 70% of the corresponding consensus sequence. If the subject sequence exhibited significant similarity to more than one consensus protein, we considered only the highest-scoring match. The family members were aligned together with the consensus protein sequence using GeneWise [36].

Families with more than two genes were defined as multigene families. In the 626 multigene families, we identified 3824 gene pairs; a gene pair is defined as one gene and its closest homolog in the same family. We defined high-similarity (HS) genes as those with a homolog in *Arabidopsis *and low-similarity (LS) as those without. LS genes and HS genes were identified based on tblastn searches using an E-value threshold of 10^-7^as previously reported [16], involving at least 50% of a given *Arabidopsis *protein or 100 amino acids. The cDNAs were aligned to protein sequences also using GeneWise. Those cDNAs containing multiple stop codons or frame-shift mutations were considered to be pseudogenes.

### Detection of Gene Conversion Events

The aligned sequences within the multigene families were used to detect conversion events using the GENECONV program version 1.81 [37]. This program detects pairs of sequences that share unusually long stretches of similarity in regions of overall lower similarity [38]. The methods used by GENECONV make it difficult to detect conversion events as candidate region lengths approach zero, i.e., for very short sequences. For example, if a conversion event contains only 3 bp of information, we cannot confidently assert the criterion of unusually long stretches of similarity, a signature of gene conversion. Both global and pairwise P-values were calculated based on 1000 permutations of original data and a BLAST-like searching algorithm.

We only used P-values from global fragments, which were multiple comparisons corrected for all possible sequence pairs. The g2 option provided by GENECONV was used to allow some accumulation of mutations in conversion regions following a candidate conversion event. With the g2 option, all multigene families that had global P-values less than 0.05 for inner fragments were considered as statistical significance requirement for conversion events; 215 of the detected pairs had P-values less than 0.01, and 162 had with P-value higher than 0.01 but less than 0.05. The inner fragment indicates a possible gene conversion event between ancestors of the two sequences in the alignment where the outer fragments have diverged.

The lengths of the converted regions were also determined using the multiple-sequence alignment used by GENECONV. We removed the gaps involved in the conversion regions of both paralogs to compensate for multiple-sequence alignment effects thereby improving the length estimate for the conversion regions. The lengths were also checked manually against pairwise alignments using randomly selected pairs without a significant difference with our multiple-sequence alignment analysis.

We compared all paralogs in each gene family. If the paralogs of a family shared more than 95% similarity, we only considered one of them as a conversion partner with other high-scoring alignments designated as members of the gene family. The goal of this step was to eliminate conversion copies resulting from subfamilies with recent duplications.

### Similarity, direction, distance and evolutionary selection analysis

The similarities between gene pairs were estimated based on their cDNA sequences. Sequence alignments and similarity scores were calculated between all paralogs within each multigene family. The genes were mapped onto the genome sequence using TBLASTN; direction and distance between gene pairs were determined from the physical map. The expected number of genes involved in conversion events on each chromosome was estimated based on the length of chromosomes. In computing sequence similarity, we compared the sequences with and without the identified converted region to exclude the contribution of converted sequence to the overall score.

Ka and Ks values of gene pairs were calculated using the LPB93 method [38]. Ka/Ks ratios were compared between gene pairs for gene-conversion partners consisting of 3824 pairs in 626 multigene families. To increase the calculation sensitivity, gene pairs with Ks > 1 were removed because the Ka/Ks calculations by LPB93 may not be reliable for highly diverged gene pairs. Functional information for gene families involved in gene conversion were obtained from NCBI [39].

## Abbreviations

LS, low similarity to *Arabidopsis; *HS, high similarity to *Arabidopsis; *IRGSP, International Rice Genome Sequencing Project.

## Authors' contributions

SX and HZ conducted the analysis. SX, HZ, SV and TC wrote the manuscript. GKSW and RL participated in the data analysis. SX, HZ, JW and XZ designed the study. All authors read and approved the final manuscript.

## Supplementary Material

Additional file 1 Annotation of genes involved in conversion events in rice genome. Detailed information of the 377 gene conversions in the rice genome including accession number, genomic location, length, direction and functional annotation. Click here for file
